# Should psychiatrists write fiction?

**DOI:** 10.1192/bjb.2017.5

**Published:** 2018-04

**Authors:** Henry Bladon

**Affiliations:** 1 Department of Creative Writing and Film Studies, University of Birmingham, Birmingham, UK

## Abstract

This paper looks at the relationship between fiction and psychiatry. Specifically, the idea of psychiatrists as fiction writers is explored, and reference is made to various fictional texts to illustrate the problems of stigma and negative imagery. These two main areas of focus are highlighted as ones that the practice of writing fiction might address, and some potential pitfalls are discussed. The paper suggests how psychiatrists might ameliorate the present problems by incorporating their unique clinical skills and knowledge into fictional narratives.

**Declaration of interest:**

None.

In 2003, Allan Beveridge examined the idea of whether psychiatrists should read fiction.^1^ This paper asks the related question of whether psychiatrists should *write* fiction? Chekhov did it, and so did Oliver Sacks, even if he did burn his first efforts. Femi Oyebode^2^ notes the similarity in the tasks of the fiction writer and the psychiatrist, pointing out that they are both trying to make sense of human behaviour. This observation is pertinent to this paper, which will look at the connection between psychiatrists and fiction writing. Certainly, fiction can be powerful, but so can psychiatry. Garcia-Nieto^3^ says that there used to be a strong connection between literature and psychiatry, a connection that was severed during the 20th century. Psychiatrist/writers can help to restore that link.

Novelists write about the human condition, that much is evident. As Baker *et al*^4^ note, for writers ‘madness has long been, and remains, a compelling preoccupation’. Fiction writers often write about psychiatry for dramatic effect, but not always faithfully. The result can produce stereotypical depictions of mental health and of the people who work in that arena. Writers also write about psychiatrists, often with the same result.

Patrick McGrath has done both; he has written about psychiatry and the experience of mental suffering (*Spider*), but he has also written about psychiatrists. In *Trauma*, his character, Charlie Weir, is a psychiatrist struggling to come to terms with his mother's death.

McGrath has also combined the two. In *Asylum,* set in a secure hospital in the late 1950s, McGrath writes about the experience of suffering, and about a young psychiatrist and his family.^5^^–^^7^

Drawing on experience is something that every writer does, but some knowledge of psychiatry is important for such genre writing. I want to suggest that writing fiction, as an avenue open to psychiatrists, presents a great opportunity to provide positive models of mental health. The understanding psychiatrists have for their patients is likely to encourage a more complete illustration of character and setting. So the answer to the question posed by this paper should be yes. The writing process essentially involves three stages. First, the writer gathers information through research. Second, a story framework, however loose or rigid that may be, is devised. Third, a coherent narrative is constructed through putting the words on to paper. When it comes to writing about psychiatry, it is plain that psychiatrists have a head start. Step one, where Sebastian Faulks, for example, spent 5 years researching his epic book about psychiatry, *Human Traces,* can be leap-frogged by psychiatrists. Similarly, the difficulty that Patrick McGrath^8^ talks about in drawing from psychiatry for fiction is largely circumvented by the closeness of psychiatrists to their subject matter and their ability to make sense of human experience.

Before the reader dashes away from the clinic armed with a notebook stuffed with observations and ideas for their new novel, I should introduce something by way of balance. The critics would suggest, for example, that somebody working therapeutically should not write, or that they may end up manipulating patients.^9^ Those in the ‘no they shouldn't write’ camp might also caution against the danger of clinical narrow-mindedness and an overkill of technical psychiatric jargon. This pitfall will be examined below. For the moment, it should be sufficient to note that the skill of a writer is in creating a world *apart* from real-world experience. Paradoxically, though, this subtle shift from fact to fiction has the aim of *seeming* real. I therefore suggest that, beyond wishing to produce an entertaining product, psychiatrists should have two principal goals in mind when approaching the writing of fiction. These are:

• correcting stigma by raising awareness, educating others and promoting good mental health
• countering the negative image of psychiatry and the psychiatrist.

## Raising awareness

The impact of media representations has major effects on the stigma experienced in mental health. That media portrayals of mental health and mental health professionals influence public perception is a well-documented phenomenon.^10^^,^^11^ As Stuart^12^ notes, the media has a ‘vast store’ of negative images in relation to mental illness.

As Liam Clarke said, literature's take on human behaviour ‘surpasses the descriptions of social and psychological sciences’.^13^ The potential power of the combination of fictional narrative and psychiatric insight could offer a potent formula of truth and wisdom resulting in enlightened understanding. The demystification of mental health services through the normalising effect of honest and informed writing cannot be understated. As Oyebode^14^ suggests, these narratives influence ‘how wider society perceives mental illness’. It therefore follows that sensitive portrayals of psychiatry would encourage a more informed and sympathetic view of the work done by psychiatrists. Rightly or wrongly, psychiatry has always endured its critics. Many of these issues are still current, whether they relate to concerns about paternalism,^15^ over-reliance on medication,^16^ the use of ‘specialist’ knowledge in the maintenance of power,^17^ criticisms of biological models,^18^ or even debates about the value of diagnostic labels.^19^^,^^20^ Psychiatrists might, therefore, wish to respond to such commentary through writing fiction that points to a more eclectic nature in the profession and what they do.

The acknowledgement that psychiatrists can move to a more collaborative relationship with the service user movement^21^ would suggest one area where fiction could develop a stronger image for psychiatrists and patients alike. Such efforts are starting to show potential, as in the recent novel by Monica Starkman, *The End of Miracles*.

The other thing to bear in mind is that although fiction may be the poor relation to the medical textbook,^22^ fiction is more accessible, and people are more likely to read a fictional account than a research paper or the DSM-5.

## Correcting the image

Psychiatry has had its periods of self-doubt. In fact, it would be hard not to see the problem as a perennial identity crisis.^23^^–^^25^ Fiction does little, it seems, to help. In her study of media representations of psychiatrists, Jacqueline Hopson claims that psychiatrists are ‘demonised’ by fiction. She asserts that that psychiatrists are regarded with suspicion and fear, and that the portrayals in fiction, which are frequently related to power, are deleterious to the profession. She points to representations such as *The Snake Pit* and Antonia White's *Beyond the Glass* in support of her thesis.^26^ Psychiatrists are without doubt in a position of power, and the idea of using psychiatric treatment as a punishment is a popular theme in fiction. If we believe what we read, it is a short step from administering medication to using it to control and punish others. *The Bell Jar*, Sylvia Plath's classic book, with its references to electroconvulsive therapy (ECT), self-harm and suicide, shows us the confusion depicted in the character of Esther Greenwood, confusion which is epitomised after her first ECT session, when she remarks: ‘*I wonder what terrible thing it was that I had done*’.

In *The Trick is to Keep Breathing*, by Janice Galloway, her character, 27-year-old Joy Stone, has doubts about her psychiatrists. Joy sets out a list of general ‘lessons’ she has learned about them, including her belief that psychiatrists are ‘devious and persistent. They always win in the end’. Such portrayals have significant consequences for those who work in mental health settings, just as they do for sufferers. For psychiatrists, negative imagery can lead to problems with recruitment,^27^ difficulties with their public image,^28^ and denigration from their colleagues in other branches of medicine.^29^

With a more positive mindset, fiction can act as a beacon of truth. Andy Bickle^30^ urges that we ‘should not underestimate the importance of literary and other media representation in creating the milieu in which we work’. In the same way that social worker Freya Barrington had the aim of raising awareness of her profession in her book, *Known to Social Services*,^31^ psychiatrist Monica Starkman relates her goal of showing psychiatry and psychiatrists as they are, and not as the stereotypes portrayed in books or films.^32^ So, a prime motivator for psychiatrists might be to adopt the ‘inform and entertain’ ideal in their approach to writing fiction, and tell readers what being a psychiatrist is *really* like. As I have already said, there are plenty of fictional psychiatrists, and they are by no means universally negative, despite what Hopson claims in her paper. In his latest novel, *Where My Heart Used to Beat*, Sebastian Faulks presents Robert Hendricks, a post-war psychiatrist who is a long way from the unpleasant stereotype Hopson bemoans. Nevertheless, there is potentially a different dimension to ‘psychiatrist point-of-view’ novels written *by* psychiatrists.

As well as correcting erroneous imagery, psychiatrists might wish to portray something of the conditions in which they work. The political dimension of psychiatry has been written about before, perhaps most strikingly in *One Flew Over the Cuckoo's Nest*, but there are other books that use narrative as a form of commentary on the state of mental health or welfare services. *Poppy Shakespeare*, by Clare Allan, is set in a psychiatric day unit. This novel, which raises issues of institutional relationships and of benefits payments, is a notable example of how fiction can highlight current difficulties in service provision.

## Pitfalls

### Ethics

*The writer is a member of society and therefore has ethical and moral responsibilities. We need to take care in the construction of our own ‘make believe’ worlds*.^33^ Ethical considerations are always prevalent in psychiatry. For psychiatrists writing fiction, there are issues of confidentiality and professional sensitivity to consider. There are always those who claim that fiction is simply fiction, but Gandolfo would disagree on the basis that fiction has the power to make a difference to people's lives. He has argued that writers should ‘rigorously question both themselves and their writing’.^34^ The fiction produced by psychiatrists might be governed not only by the desire to tell a story, it will also be tempered by the need to provide honest and realistic portrayals of the subject matter. Of course, the ethical dimension can be productive in itself. Beveridge^35^ points out that writers might want to explore moral quandaries, a subject particularly prevalent in psychiatry. Issues of power, liberty, treatment and the like are fertile topics that do not have to be restricted to textbooks and non-fiction.

### ‘Medical’ fiction

Psychiatrist Monica Starkman asks whether psychiatrists write good novels. In attempting to answer her own question, she points out that psychiatrists have access to ‘the deepest, most private thoughts and feelings of many people’, which might lead one to assume that fiction writing should be easier for a psychiatrist. However, writing should be informed, but it should also be entertaining, and another obstacle for potential ‘psychiatrist fiction writers’ is technical language. Beveridge^36^ suggests that writers are attempting to do crudely what modern psychologists do in a sophisticated manner. This is missing the point of fiction, but it is a point he later seems to correct. In 2010, he said that fiction can ‘deepen our understanding of people with a mental illness’ and divert from the narrow evidence-based approach to psychiatry.^37^ Potential writers should always think about the reader, so psychiatrist/writers should resist the desire to overdo psychiatric terminology. As Crawford and Baker note,^2^,^2^ it should be remembered that fictional texts are *representations* of illnesses. They also reiterate the fact that fiction is not written for the purposes of diagnosis. To ‘diagnose or not’ is a consideration psychiatrist fiction writers will face. Oyebode^14^ points out that novels are not scientific studies of psychopathology; in a discussion I had with award-winning novelist Nathan Filer (*The Shock of the Fall*), Filer pointed out that, despite the protagonist in the novel having a strong psychotic dislocation, the term ‘schizophrenia’ was only used twice in his book. There are other writers, Nicola Barker, for instance (*Reversed Forecast*), who similarly convey a sense of suffering without resorting to psychiatric terminology.

## Conclusion

Psychiatrists are dealt a rough hand by fiction. Despite more light-hearted depictions such as the satirical archetype produced by Will Self in a number of his novels (Dr Zack Busner appears in a succession of Self's novels and short stories, *The Quantity Theory of Insanity*, *Ward 9*, *Dr Mukti*, *Umbrella*, *The Book of Dave*, *Shark*), the work of the psychiatrist is depicted as mysterious at best and threatening at worst. Such imagery is only likely to have negative results. Not only does this affect the profession, but it filters down to the end-users of mental health services.

Although Bickle^30^ suggests that fiction deals with the ‘wrong type of data’ to convey a comprehensive knowledge of mental illness, Bickle^30^ makes the point that it offers the chance to step back from the world and to explore these issues, thereby helping psychiatrists reflect on their practice. The efforts of psychiatrist/writers in producing fiction presents an opportunity to correct some of these ills. If psychiatrists can avoid the pitfalls, then writing fiction can achieve a number of positive outcomes.

## About the author

**Henry Bladon**, Final-year PhD student at the University of Birmingham in the Department of Creative Writing and Film Studies, Birmingham, UK.
